# Whole-Genome Analysis of *Bartonella* *ancashensis*, a Novel Pathogen Causing Verruga Peruana, Rural Ancash Region, Peru

**DOI:** 10.3201/eid2303.161476

**Published:** 2017-03

**Authors:** Kristin E. Mullins, Jun Hang, Robert J. Clifford, Fatma Onmus-Leone, Yu Yang, Ju Jiang, Mariana Leguia, Matthew R. Kasper, Ciro Maguina, Emil P. Lesho, Richard G. Jarman, Allen Richards, David Blazes

**Affiliations:** Uniformed Services University of the Health Sciences, Bethesda, Maryland, USA (K.E. Mullins, J. Jiang, A. Richards, D. Blazes);; US Naval Medical Research Center, Silver Spring, Maryland, USA (K.E. Mullins, A. Richards);; Walter Reed Army Institute of Research, Silver Spring (J. Hang, R.J. Clifford, F. Onmus-Leone, Y. Yang, E.P. Lesho, R.G. Jarman);; US Naval Medical Research Unit No. 6, Lima, Peru (M. Leguia, M.R. Kasper);; Universidad Peruana Cayetano Heredia, Lima (C. Maguina)

**Keywords:** Bartonella, Bartonella ancashensis, Bartonella bacilliformis, bacteria, bartonellosis, novel phylogenetic lineage, whole-genome analysis, verruga peruana, rural Ancash region, Peru

## Abstract

The genus *Bartonella* contains >40 species, and an increasing number of these *Bartonella* species are being implicated in human disease. One such pathogen is *Bartonella ancashensis*, which was isolated in blood samples from 2 patients living in Caraz, Peru, during a clinical trial of treatment for bartonellosis. Three *B. ancashensis* strains were analyzed by using whole-genome restriction mapping and high-throughput pyrosequencing. Genome-wide comparative analysis of *Bartonella* species showed that *B. ancashensis* has features seen in modern and ancient lineages of *Bartonella* species and is more related to *B. bacilliformis*. The divergence between *B. ancashensis* and *B. bacilliformis* is much greater than what is seen between known *Bartonella* genetic lineages. In addition, *B. ancashensis* contains type IV secretion system proteins, which are not present in *B. bacilliformis*. Whole-genome analysis indicates that *B. ancashensis* might represent a distinct *Bartonella* lineage phylogenetically related to *B. bacilliformis*.

Bartonelloses are major emerging infectious bacterial diseases because of the high prevalence of chronic *Bartonella* infections in mammals and humans and their increasing risk for infection of immunocompromised populations (*1*–*4*). *Bartonella* species are present worldwide and are associated with several diseases, such as Carrion’s disease, caused by *B. bacilliformis*; cat-scratch disease, caused by *B. henselae*; and trench fever, caused by *B. quintana* (*2**,**5*–*7*). *B. henselae* and *B. quintana* are also associated with chronic infections, such as bacillary angimatosis and endocarditis, and *B. quintana* chronic bacteremia is found in homeless populations worldwide (*3*,*8*–*12*).

In addition to these 3 major infectious pathogens, an increasing number of new *Bartonella* spp. have been identified in recent years as zoonotic pathogens transmitted by diverse arthropod vectors (*1*,*2*,*7*). Currently, >40 official and candidate *Bartonella* species are listed in the Taxonomy Database of the National Center for Biotechnology Information (http://www.bacterio.net/Bartonella.html); 31 are recognized species. During their evolution, *Bartonella* lineages have adapted to a variety of hosts and developed virulence factors associated with a diverse set of disease signs and symptoms (*13*–*15*).

Despite the high mortality rate for bartonellosis in Peru, studies on *Bartonella* pathogens are insufficient and mainly focused on *B. bacilliformis* (*16*). We previously reported identification of non-*bacilliformis Bartonella* in Peru during a clinical treatment trial (*17*–*19*). Using single-locus sequence typing, we identified 3 isolates (designated 20.00, 20.60, and 41.60) from 4 whole blood specimens collected from 2 patients (nos. 20 and 41) at day 0 or 60 during the clinical treatment trial in the Ancash region of Peru and found that these isolates had citrate synthase (*gltA*) gene sequences that diverged from that of *B. bacilliformis* reference strain KC583 (*17*–*19*). Multilocus sequencing typing and microbiological analyses indicated these 3 isolates are members of a novel *Bartonella* species, subsequently name *B. ancashensis* (*17*).

In this study, we performed genomic analyses of 3 non-*bacilliformis Bartonella* isolates obtained from patient blood samples (isolates 20.00, 20.60, and 41.60). Whole-genome analyses confirmed our previous identification of the isolates as a new species (*B. ancashensis*) (*20*) and identified unique genomic characteristics of *B. ancashensis* and differences between *B. ancashensis* and its closest relative (*B. bacilliformis*).

## Methods

### Ethics Statement

The human subject use protocol, including clinical specimen collection, and the consent procedure were approved in 2002 by the Institutional Review Boards of the Uniformed Services University of the Health Sciences (Bethesda, MD, USA); the Naval Medical Research Center Institutional Review Board (Bethesda, MD, USA); and the Universidad Peruana Cayetano Heredia (Lima, Peru). The trial details are available in the International Standard Randomized Controlled Trial Number registry (https://www.isrctn.com; trial no. ISRCTN16597283). Patients enrolled were 1–60 years of age, and written consent was obtained from the patient or the parent or guardian of the patient enrolled in the study.

### Study Protocol

A clinical trial to compare rifampin, the standard drug for treatment of bartonellosis caused by chronic *B. bacilliformis* infection, with azithromycin, a possible alternative drug, was conducted in 2003 in the Caraz District of the Ancash region of Peru (Blazes DL, trial no. ISRCTN16597283). Patients with suspected chronic *B. bacilliformis* infection (verruga peruana) either came to the local hospital in Caraz or were identified by home visits. Patients (>1 year of age) with verruga peruana were randomly chosen to receive either a daily dose of rifampin (Pfizer, New York, NY, USA) for 14 days or 2 weekly doses of azithromycin (Pfizer) on days 0 and 7. For the patients who participated in the trial, survey data and medical records were collected on day 0 (baseline; time of presentation at the local hospital), and patients were then given the 2-week antimicrobial drug treatment. Clinical data was also collected on days 7, 14, 30, and 60.

In addition, peripheral blood specimens were collected from each patient on days 0, 7, 14, and 60 into tubes containing sodium citrate solution at the local hospital in Caraz and transported on ice to the clinical laboratory at the US Naval Medical Research Unit No. 6 (Lima, Peru) for blood cultures and analysis by PCR. Selected specimens, *Bartonella* isolates, and genomic DNA extracts were sent to the Naval Medical Research Center (Silver Spring, MD, USA) and the Walter Reed Army Institute of Research (Silver Spring, MD, USA) for additional investigations.

Blood specimens were cultured for >8 weeks as described (*17*–*19*). *Bartonella* culture-positive specimens were confirmed to be *Bartontella* species by using microbiological observations and molecular assays. Nucleic acids were isolated from culture-positive blood samples and subjected to PCR amplification of a 338-bp fragment of the *gltA* gene. The PCR product was sequenced by using the Sanger method.

We aligned partial *gltA* gene sequences (homologous to nt 781–1137 of the *B. bacilliformis* KC583 *gltA* gene) and used them for phylogenetic analyses. Samples with *gltA* sequences that showed major differences (>85% divergence) from those of *B. bacilliformis* were cultured on brain heart infusion agar supplemented with 10% defibrinated sheep blood (BD Diagnostics, Glencoe, MD, USA) for 10–28 days at the Naval Medical Research Center.

For next-generation sequencing (NGS), genomic DNA was extracted from *Bartonella* isolates, randomly fragmented by using focused ultrasonication (S2 System; Covaris, Inc., Woburn, MA, USA), and used in rapid shotgun genomic DNA library preparation and pyrosequencing with the 454 GS FLX Titanium System (Roche 454 Life Sciences, Branford, CT, USA). For whole-genome restriction map (WGRM) analysis, *Bartonella* isolates were freshly grown on brain heart infusion agar with 10% sheep blood at 30°C in an atmosphere of 5% CO_2_ for 10–14 days. High molecular weight DNA was isolated by using the Argus Sample Preparation Kit (OpGen Inc., Gaithersburg, MD, USA). DNA quality and quantity were assessed by using the Argus QCard Kit and mapped by using the Argus MapCard Kit and Argus Enzyme Kit-AflII with the Argus System (OpGen Inc.). Downstream analysis, clustering, and genome alignment was performed by using MapSolver version 3.2.4. (OpGen Inc.).

We de novo assembled NGS data into sequence contigs by using GS Assembler software version 2.5.3 (Newbler; https://wikis.utexas.edu/display/bioiteam/GS+De+novo+assembler) and then assembled the contigs to scaffolds with the WGRM as the physical reference. PCR amplification and open reading frame annotation were used to complete genome assembling. After we virtually digested the complete genome sequence with *Afl*II, we aligned the in silico whole-genome restriction map with the WGRM to ensure the correct order and orientation of the final assemblies (*21*).

We performed pairwise genome-wide comparative analysis by comparing homologous proteins from pairs of *Bartonella* species. Results are presented as density distribution curves for amino acid identity and as dot plots for pairwise amino acid identity for each homologous protein. Genes of interest were subjected to additional analysis, including gene cluster comparisons. For genome-wide phylogenetic analysis of *Bartonella* species, complete genome sequences or assembly contigs of whole genome sequences were aligned by using Mauve version 2.3.1 (*22*) to identify single nucleotide changes in conserved genomic regions. A total of 12,740 single-nucleotide polymorphisms were found in in 25.2 kb of sequence common to all 38 *Bartonella* strains. A phylogenetic tree was constructed by using the R phangorn package (*23*). The initial tree was constructed by using the neighbor-joining algorithm and optimized by using the parsimony maximum-likelihood method. Tree stability was evaluated by using 100 bootstrap replicates.

## Results

In the clinical trial testing the efficacy of rifampin and azithromycin for treatment of chronic bartonellosis, blood specimens from 72 of 127 patients were positive for *Bartonella* species by culture, and *gltA* gene sequencing indicated that these patients were infected with only *B. bacilliformis*; however, 2 patients (nos. 20 and 41) were infected with *B. ancashensis* (*17*–*19*). DNA extracts from the 4 original whole blood specimens from these patients (20.00, 20.60, 41.00, and 41.60) were tested by using quantitative bacterial 16S rDNA PCR, standard bacterial 16S PCR, and *B. ancashensis*–specific PCR (Table 1). Whole blood from patient 20 was PCR negative for bacteria on days 0 and 60. However, high levels of bacteremia were seen for whole blood specimens from days 0 and 60 for patient 41.

**Table 1:** Characteristics of 4 non-*bacilliformis Bartonella* isolates from 2 patients with verruga peruana, rural Ancash region, Peru*

Characteristic	**Patient 20, 3-y-old boy, isolate no.**		**Patient 41, 10-y-old boy, isolate no.**	
	20.00	20.60	41.00	41.60
Patient signs	Lesions on hands and feet that disappeared after antimicrobial drug treatment		Lesions on hands and feet that disappeared after antimicrobial drug treatment	
Antimicrobial drugs used	Azithromycin on days 0 and 7		Rifampin daily on days 0–14	
Whole blood collection time	Day 0	Day 60	Day 0	Day 60
Peripheral blood smear†	Negative	Negative	Negative	Negative
Blood culture for *Bartonella *sp.	Positive	Positive	Positive	Positive
16S 321/533 TaqMan qPCR‡	Negative	Negative	3.93 × 10^5^ (19.24)	6.36 × 10^4^ (22.15)
16S 27F2/533R PCR	Negative	Negative	Positive	Positive
*B. ancashensis*–specific PCR§	Negative	Negative	Positive	Positive
Blood culture *gltA *PCR/sequencing	*B. ancashensis*	*B. ancashensis*	B. *bacilliformis*	*B. ancashensis*
Isolate by pure-culture sequencing	*rrs*, *gltA*, *rpoB*; whole genome	*rrs*, *gltA*, *rpoB*; whole genome	*gltA*	*rrs*, *gltA*, *rpoB*; whole genome

*gltA, citrate synthase gene; *rpoB*, β subunit of RNA polymerase gene; *rrs*, 16S rRNA gene.  †Rapid microscopic diagnosis for Carrion’s disease (*16*). ‡Values are 16S rRNA gene copy number/microliter (quantitative cycle threshold). §Primers 22RC-3F (5′-TTCGGCTTAGCTTATCCGTTTCACAA-3′) and 32RC-5R (5′-CGTAAGAGCTTTGTGGCAAAATAGCAA-3′) were used; the expected PCR amplicons size was 0.8 kb, which corresponds to nt 673839–674636 of *B. ancashensis* (GenBank accession no. CP010401).

Although levels of bacteremia differed greatly, clinical signs and symptoms for both patients were indistinguishable from each another and from those for other patients with confirmed cases of chronic *B. bacilliformis* infection. In addition, our results confirm that *B. ancashensis* was isolated from whole blood specimens of patient 41 on day 60, but not on day 0. The evidence suggests emergence of a novel *Bartonella* species in Peru that can cause its own verruga peruana–like infection in humans or possibly co-infect humans in conjunction with *B. bacilliformis*. It is intriguing that the bacteremia profile, based on blood cultures, for patient 41 changed from *B. bacilliformis* at day 0 to *B. ancashensis* at day 60.

**Figure 1:**
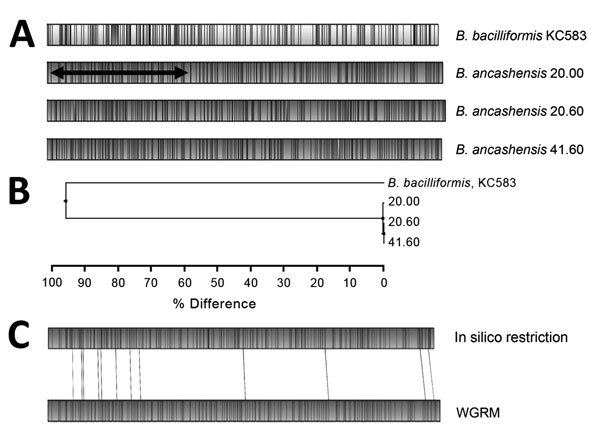
Alignment of whole-genome restriction maps and in silico map for *Bartonella* isolates from patients with verruga peruana, rural Ancash region, Peru. A) Maps for *B. ancashensis* isolates 20.00, 20.60, and 41.60 were determined by using optical mapping. Shaded areas indicate regions of alignment, unshaded areas indicate regions where restriction maps do not align, and black horizontal arrow indicates restriction sites. B) Phylogeny based on map similarity constructed by using the unweighted pair group method with arithmetic mean. C) Alignment of map for *B. ancashensis* predicted in silico from the complete genome sequence with the experimentally observed *B. ancashensis* map. WGRM, whole-genome restriction map.

We performed genome-wide analysis of the 3 *B. ancashensis* isolates (20.00, 20.60, and 41.60) by using the WGRM and NGS (*21*,*24*). The WGRMs of the 3 isolates showed >99.7% similarity with each other and <10% similarity to the WGRM of *B. bacilliformis* KC583. WGRM showed that the *B. ancashensis* genome is circular and ≈1.46 Mb. A region of ≈0.64 Mb in the 20.00 genome was inverted when compared with maps for isolates 20.60 and 41.60 (Figure 1).

The complete genome sequence of *B. ancashensis* strain 20.00 (GenBank accession no. NZ_CP010401) is circular (1,466,048 bp) and has a G + C content of 38.4%. These values are similar to those for the complete genome sequence of *B. bacilliformis* KC583 (NC_008783.1) (1,445,021 bp and a G + C content of 38.2%). The inverted region of the 20.00 genome (0.64 Mb) is flanked by two 507-bp repetitive sequences in an opposite orientation. The repetitive sequence is 97% identical to the gene for *B. bacilliformis* integrase (protein family HMM PF00589), a DNA breaking–rejoining enzyme that catalyzes DNA recombination.

**Figure 2:**
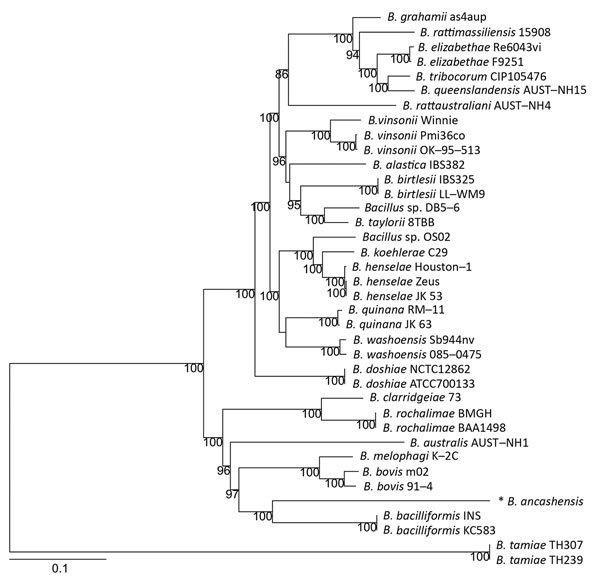
Phylogenetic relationship of *Bartonella ancashensis* isolates from patients with verruga peruana, rural Ancash region, Peru, with other *Bartonella* species based on whole-genome phylogeny. The tree is based on single-nucleotide polymorphisms identified in genomic regions common to all *Bartonella* strains examined. The initial tree was constructed by using the neighbor-joining algorithm and was optimized by using the parsimony maximum-likelihood method. Tree stability was evaluated by using 100 bootstrap replications. Asterisk indicates strain isolated in this study. Numbers along branches are bootstrap values. Scale bar indicates nucleotide substitutions per site.

**Figure 3:**
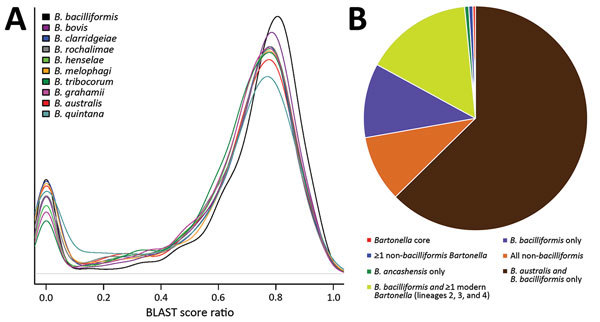
Proteomic analysis of *Bartonella ancashensis* isolated from patients with verruga peruana, rural Ancash region, Peru, and 10 related *Bartonella* species. A) Density plot showing similarity between *B. ancashensis* protein-coding genes and genes from 10 of the more closely related *Bartonella* species. Similarity scores are based on the BLAST score ratio method (BSR) (*25*). A score of 1.0 indicates identity between 2 proteins, and a score <0.3 indicates that the 2 proteins do not show meaningful similarity. The *Bartonella* species whose protein similarity score distribution has a peak closest to 1 (*B. bacilliformis*) has the highest overall protein similarity to *B. ancashensis*. Horizontal gray line indicates density = 0. B) Presence of homologous protein-coding genes in *B. ancashensis* and 15 *Bartonella* species. Proteins from 2 species with a BSR score >0.3 were considered to be homologs and present in *B. ancashensis* and >1 *Bartonella* species.

We used whole-genome phylogenetic analyses to examine the evolutionary relatedness between *B. ancashensis* and 40 other *Bartonella* strains (Table 2; Figure 2). This analysis, based on 12,740 single-nucleotide polymorphisms in genomic sequences conserved across all species, showed that *B. ancashensis* isolate 20.00 is most closely related to *B. bacilliformis*, *B. bovis*, and *B. melophagi*. The degree of similarity between *B. ancashensis* and 15 other *Bartonella* species was further examined by assessing the pairwise similarity of homologous protein with the basic local alignment search tool score ratio method (*25*) (Figure 3, panel A). *B. ancashensis* predicted proteins are most similar to *B. bacilliformis* proteins, but there are several distinctive differences. Specifically, 63.7% of *B. ancashensis* genes are part of a core genome common to all 15 comparator species, 10.7% of *B. ancashensis* proteins did not have a homolog in any of the reference *Bartonella* spp., and 89.3% of *B. ancashensis* proteins are present in <1 other *Bartonella* spp.; 79.1% of *B. ancashensis* proteins were found in *B. bacilliformis* (Figure 3, panel B).

**Table 2:** *Bartonella* spp. strains (n = 41) used for whole-genome analysis of human pathogens causing verruga peruana, rural Ancash region, Peru*

Species	Strain	GenBank accession no.
*B. alsatica *	IBS 382	AIME01000000
*B. ancashensis*	20.00	NZ_CP010401
*B. australis*	Aust/NH1	NC_020300.1
*B. bacilliformis*	INS	AMQK01000000
	KC583	NC_008783.1
*B. birtlesii*	IBS 325	AKIP01000000
	LL-WM9	AIMC01000000
*B. bovis*	91-4	AGWA01000000
	m02	AGWB01000000
*B. clarridgeiae*	73	NC_014932.1
*B. doshiae*	NCTC 12862	AILV01000000
	ATCC 700133	JAGY01000000
*B. elizabethae*	F9251	AIMF01000000
	Re6043vi	AILW01000000
*B. grahamii*	as4aup	CP001562.1
*B. henselae*	Houston-1	BX897699.1
	JK 53	AHPI01000000
	Zeus	AHPJ01000000
*B. koehlerae*	C29	AHPL01000000
*B. melophagi*	K-2C	AIMA01000000
*B. queenslandensis*	AUST/NH15	CALX01000000
*B. quintana*	JK 31	AHPG01000000
	JK 63	AHPF01000000
	JK 67	AHPC01000000
	JK 68	AHPD01000000
	RM-11	CP003784.1
*B. rattaustraliani*	AUST/NH4	CALW02000000
*B. rattimassiliensis*	15908	AILY01000000
*B. rochalimae*	ATCC BAA-1498	FN645455.1–FN645467.1
	BMGH	AHPK01000000
*Bartonella *sp. DB5-6	DB5-6	AILT01000000
*Bartonella *sp. OS02	OS02	CALV01000000
*B. tamiae*	Th239	AIMB01000000
	Th307	AIMG01000000
*B. taylorii*	8TBB	AIMD01000000
*B. tribocorum*	CIP 105476	AM260525.1
*B. vinsonii*	OK-94-513	AILZ01000000
	Pm136co	AIMH01000000
	Winnie	NC_020301.1
*B. washoensis*	085-0475	AILX01000000
	Sb944nv	AILU01000000

*NA, not available.

Most *B. ancashensis* genes identified by pan-genome analyses had homologs in >1 *Bartonella* species (Figure 3, panel B). Eight protein-coding genes in *B. ancashensis* and *B. bacilliformis* were not present in any other species. An additional 5 protein-coding genes were present in *B. ancashensis*, *B. bacilliformis*, and *B. australis*, but in no other known species. In contrast, there were 8 protein-coding genes in *B. ancashensis* and all 14 species, excluding *B. bacilliformis*, but including *B. australis*, which is considered the most divergent *Bartonella* species currently recognized. Another 129 *B. ancashensis* protein-coding genes have homologs in only a subset of the non-*bacilliformis Bartonella* (*26*).

**Figure 4:**
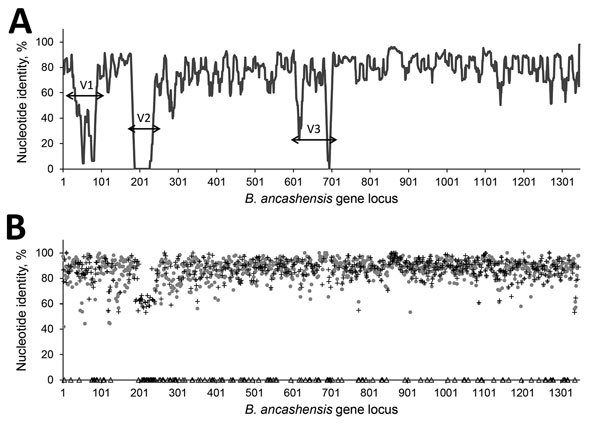
Pairwise comparisons of protein-coding genes of *Bartonella* isolates from patients with verruga peruana, rural Ancash region, Peru. A) Nucleotide similarity of *B. ancashensis* protein-coding sequences compared with those of *B. bacilliformis* (gray circles in panel B), top hit non-*bacilliformis Bartonella* (+ in panel B) and not determined hypothetic proteins (Δ in panel B). B) Nucleotide similarity plot. Average nucleotide identity within a window of 10 genes was plotted against gene locus number. Regions I, II, and III are 3 variable regions that contain genes with lower nucleotide identities or absent in the *B. bacilliformis* genome. V, variable.

In 3 regions of the *B. ancashensis* genome, the similarity between *B. bacilliformis* and *B. ancashensis* proteins was lower than the average value (Figure 4). Proteins in variable region 1 (genes 30–90) are homologous to non-*bacilliformis* species proteins, including phage proteins, such as HigA and HigB. Proteins encoded in variable region 2 (genes 180–240) are absent from *B. bacilliformis.* Among these proteins are those that have high similarity with *Bartonella* type IV secretion system proteins, which are hypothetical gene products that have moderate identity with proteins from other non-*bacilliformis* species, and novel hypothetical proteins. Variable region 3 (genes 620–704) contains loci encoding hypothetical proteins not seen in other species and several toxin proteins that are not found in *B. bacilliformis*, including the RelE/StbE replicon stabilization toxin, the RelB/StbD replicon stabilization protein, and the HigB toxin protein.

**Figure 5:**
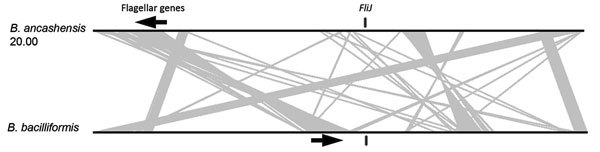
Genetic arrangement of the genome of *B. ancashensis* isolate 20.00 from a patient with verruga peruana, rural Ancash region, Peru, compared with that of *B. bacilliformis* KC583. Black lines indicate chromosomes and gray lines link syntenic genomic regions that are rearranged between the 2 genomes. *FliJ* genes are indicated by black vertical bars, and flagellar gene clusters are indicated by arrows, which indicate direction of transcription.

Analysis identified 2 characteristic features of *B. ancashensis*: type IV secretion complex (VirB2) proteins, which are not found in *B. bacilliformis*; and flagella proteins, which are not found in *Bartonella* species in lineage 4, including human pathogens *B. quintana* and *B. henselae* (*15*,*27*,*28*). In isolates 20.60 and 41.60, the 31 flagellar genes encoded by *B. ancashensis* are located in the identical order and distances as their homologs in the *B. bacilliformis* genome. Isolate 20.00 has a large genomic inversion, and this rearrangement results in 1 gene (*FliJ*) required for production of flagella arranged in a reversed orientation and separated from the other genes of the main flagellar gene cluster. In isolate 20.00, *FliJ* is ≈600 kb from the flagellar gene cluster; in isolates 20.60 and 41.60, *FliJ* is ≈100 kb from this cluster (Figure 5).

**Figure 6:**
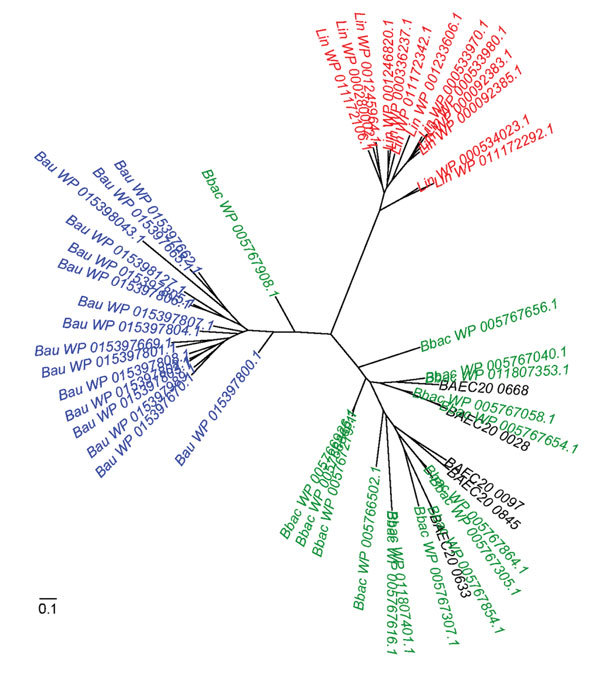
Virulence-modulating (VM) proteins in *Leptospira* and *Bartonella* species. Unrooted phylogenetic tree of VM proteins from *Leptospira interrogans* (*Lin*, red), *B. australis* (*Bau*, blue), *B. bacilliformis* (*Bbac*, green), and *B. ancashensis* (*BAE*, black). VM proteins from *L. interrogans*, *B. bacilliformis*, and *B. australis*, cluster by species; the 5 VM proteins encoded by *B. ancashensis* group with their *B. bacilliformis* homologs. Scale bar indicates amino acid substitutions per site.

A *Leptospira* species virulence attenuation study identified a group of paralogous virulence modulated (VM) genes, which are believed to play a role in human pathogenesis caused by *Leptospira interrogans* (*29*). Subsequent comparative genomic analysis showed that VM proteins are present in other bacterial pathogens, including *B. bacilliformis* and *B. australis*; *B. ancashensis* encodes 5 VM proteins (Figure 6). In contrast, no homologs of VM proteins were found by a basic local alignment search tool search in any other recognized *Bartonella* species. As seen for the VM proteins of *Leptospira* species, VM protein genes in these 3 *Bartonella* species were scattered throughout their genomes, and the number of VM proteins was different for each species.

## Discussion

*B. bacilliformis* was discovered in Peru in 1907 as the causative agent for Oroya fever and verruga peruana. Since then, *B. bacilliformis* has been the primary subject in bartonelloses studies in South America. However, it has long been speculated that multiple *Bartonella* spp. might be circulating in this region (*30*). Our study and previous work clearly indicate *B. ancashensis* is a unique *Bartonella* species that co-circulates with *B. bacilliformis* in the Ancash region of Peru, where the prevalence of *B. bacilliformis* is high (*16*,*31*,*32*).

Moreover, *B. ancashensis* has several unique genomic features. Like *B. bacilliformis*, this species encodes flagellar genes, which are believed to be essential for erythrocyte invasion, the main route of pathogenesis caused by *B. bacilliformis* (*33*). In contrast, human pathogens *B. quintana* and *B. henselae* do not produce flagella, and host-specific pathogenicity is believed to be linked to the type IV secretion system (T4SS) gene clusters VirB and Trw (*27*,*28*,*33*,*34*). *B. ancashensis* and 2 other pathogens (*B. clarridgeiae* and *B. rochalimae*) have flagellar and VirB T4SS gene clusters (*15*,*26*,*27*).

Whole-genome analysis of 3 *B. ancashensis* isolates showed that isolate 20.00 differs from the other 2 isolates by a large genomic region inversion. Future comparative analysis of gene expression profiles in these strains will show whether this genomic inversion alters the regulation of flagellar genes, as well as other virulence factors within or adjacent to the inverted region.

Moreover, it will be useful to investigate whether the genomic inversion (isolate 20.00 vs. 20.60) is related to administration of antimicrobial drugs to infected patients because isolates 20.60 and 41.60 were obtained from patients after they received antimicrobial drugs and cleared any clinical signs or symptoms of bartonellosis because infection persisted 60 days after enrollment in the study and administration of antimicrobial drugs. Furthermore, these isolates were previously tested for their in vitro susceptibility to rifampin and azithromycin and both were found to be susceptible (*17*). However, in vitro antimicrobial drug susceptibility testing for *Bartonella* spp. has been largely limited in its clinical utility. However, *B. ancashensis* might be capable of producing chronic asymptomatic infections that could be caused by its unique genomic characteristics.

The VM genes belong to a family of homologous virulence-related genes originally identified in *L. interrogans* and modulate the pathogenesis of *L. interrogans* in humans (*29*). These genes in *B. bacilliformis*, *B. australis*, and *B. ancashensis*, but not in other *Bartonella* spp., the further comparative analysis and functional studies on these VM proteins and the large number of other hypothetical proteins in *Bartonella* spp. will shed light on the pathogenesis mechanisms of bartonellosis, which are so far largely unknown.

Intensified tropical disease surveillance and advances in scientific methods led to an increasing number of new *Bartonella* species being identified in recent years (*30*,*35*). These studies identified 1 major phylogenetic lineage of the genus *Bartonella*. Our study and other genomic studies demonstrated that *B. bacilliformis*, which was historically regarded as the ancestral *Bartonella* spp., probably diverged from other species in the distant past and evolved as a species uniquely adapted to the human host because no small mammals have been implicated as reservoir hosts for *B. bacilliformis* (*15*,*26*,*27*,*36*). Although *B. ancashensis* is a novel species most closely related to *B. bacilliformis*, it has a nucleotide divergence of ≈20% when compared with *B. bacilliformis* for conserved genomic regions, which is exceedingly high and comparable with distances among proposed *Bartonella* lineages (*15*,*26*). Therefore, it is rational to designate *B. ancashensis* as an independent lineage parallel to the *B. bacilliformis* lineage. Our study provided evidence that there might be more *Bartonella* species and subspecies in regions of South America.

Bartonellosis has affected humans for hundreds to thousands of years, remains endemic to several areas, and continues to cause sporadic outbreaks in many regions. Identification of a novel *Bartonella* species in this study not only provided long-awaited evidence of species diversity in areas to which *B. bacilliformis* is endemic but also indicates the need for acquisition of sufficient genomic data, which will enable pathogenomics studies. Such studies will make essential contribution to a comprehensive understanding and effective control of bartonelloses.
